# Biochemodynamic Features of Metal Ions Bound by Micro- and Nano-Plastics in Aquatic Media

**DOI:** 10.3389/fchem.2018.00627

**Published:** 2018-12-14

**Authors:** Raewyn M. Town, Herman P. van Leeuwen, Ronny Blust

**Affiliations:** ^1^Systemic Physiological and Ecotoxicological Research (SPHERE), Department of Biology, University of Antwerp, Antwerp, Belgium; ^2^Physical Chemistry and Soft Matter, Wageningen University & Research, Wageningen, Netherlands

**Keywords:** microplastic, nanoplastic, kinetics, dynamic metal speciation, bioavailability

## Abstract

A simple model, based on spherical geometry, is applied to the description of release kinetics of metal species from nano- and micro-plastic particles. Compiled literature data show that the effective diffusion coefficients, *D*_eff_, for metal species within plastic polymer bodies are many orders of magnitude lower than those applicable for metal ions in bulk aqueous media. Consequently, diffusion of metal ions in the aqueous medium is much faster than that within the body of the plastic particle. So long as the rate of dissociation of any inner-sphere metal complexes is greater than the rate of diffusion within the particle body, the latter process is the limiting step in the overall release kinetics of metal species that are sorbed within the body of the plastic particle. Metal ions that are sorbed at the very particle/medium interface and/or associated with surface-sorbed ligands do not need to traverse the particle body and thus in the diffusion-limiting case, their rate of release will correspond to the rate of diffusion in the aqueous medium. Irrespective of the intraparticulate metal speciation, for a given diffusion coefficient, the proportion of metal species released from plastic particles within a given time frame increases dramatically as the size of the particle decreases. The ensuing consequences for the chemodynamics and bioavailability of metal species associated with plastic micro- and nano-particles in aquatic systems are discussed and illustrated with practical examples.

## Introduction

Microplastics (MPs) are ubiquitous worldwide in the water column of freshwater and marine systems (Barnes et al., 2009; Baldwin et al., 2016; Leslie et al., 2017; Schmidt et al., 2017), in sediments (Blumenröder et al., 2017; Graca et al., 2017; Leslie et al., 2017; Wang et al., 2017), in soils (Scheurer and Bigalke, 2018; Zhang and Liu, 2018; Zhou et al., 2018), and within biota (Foekma et al., 2013; Goldstein and Goodwin, 2013; Lusher et al., 2015; Leslie et al., 2017; Digka et al., 2018; Piccardo et al., 2018; van der Hal et al., 2018). The size range of plastic particles denoted as being MPs generally corresponds to a diameter in the range 100 nm to 5 mm (European Food Safety Authority, 2016; Alimi et al., 2018). Current monitoring approaches for MPs in the water column employ plankton nets, and thus particles with dimensions smaller than some 10 μm are not collected. Nevertheless, strategies are emerging for detection and size characterization of nanoplastics (NPs), i.e., plastic particles with diameter in the range of order 1–100 nm (ter Halle et al., 2017). The number of NPs in the environment remains to be verified, but very high numbers of these entities could be present: in addition to primary sources, NPs will be generated by abiotic and biotic degradation of MPs. For example, UV degradation of MPs has been shown to generate NPs (Gigault et al., 2016; Lambert and Wagner, 2016a,b), and digestive fragmentation has been proposed as a means by which NPs can be rapidly generated from MPs (Dawson et al., 2018). Indeed, embrittlement of plastics by abiotic processes will likely facilitate their further degradation by biotic processes. Simply from mass conservation, one spherical particle of 10 μm radius could generate 10^9^ particles of 10 nm radius. Indeed, for MPs collected from marine systems, the reported number distributions as a function of size show that as the size decreases, the increase in abundance follows a power law (Enders et al., 2015; Erni-Cassola et al., 2017), and studies on MP degradation patterns show a similar trend (ter Halle et al., 2016). It is thus anticipated that in aquatic ecosystems the number of plastic particles in a given size fraction will increase with time from the micro to the nanoscale.

Plastic particles represent a heterogeneous class of materials in terms of their physical features (size, shape) and physicochemical properties, e.g., chemical functionality, porosity, hydrophobicity. The distribution of types of polymers found in floating and beached plastic particles reflects the release abundance and properties of the original polymers, e.g., density, as well as degradation processes, e.g., photooxidation, hydrolysis, acquisition of organic coatings. The composition of floating and beached plastics is dominated by high and low density polyethylene (HDPE, LDPE) *ca*. 50–70%, and polypropylene (PP) *ca*. 20%, plus a few percent each of polyamides (PA), polyvinyl chloride (PVC), polystyrene (PS), and poly(ethyleneterephthalate) (PET) (Constant et al., 2018; Digka et al., 2018; Falcou-Préfol et al., 2018; Suaria et al., 2018). A similar abundance distribution of polymers is found for MPs in biota sampled in aquatic ecosystems (Digka et al., 2018), suggesting that biouptake is not very selective with respect to the polymer type.

In addition to the bulk polymer, plastics contain a range of associated compounds, which generally includes diverse species of metals and organics. Such compounds include those that are inherent components of the original material, e.g., plasticizers, stabilizers, surface modifiers, flame retardants, and pigments, as well as those that are environmentally acquired by sorption from the surroundings. For the case of metals, Pb and Zn compounds are used as stabilizers in plastics, and Pb and Cd compounds are used as color pigments. The inherent metal content of plastics, on a w/w basis, can be up to *ca*. 1% Cd, *ca*. 2% Pb, and *ca*. 10% Zn. In Europe, the use of Pb and Cd additives in plastics is now restricted, but such regulations are not internationally applicable, and there are large quantities of legacy plastics in the environment. In addition to the inherent metal content, plastics can sorb metals from the environment into the polymer matrix as well as via sorbed coatings of complexing organic matter (Ashton et al., 2010; Holmes et al., 2012, 2014; Rochman et al., 2014; Brennecke et al., 2016). Several studies have documented the metal contents of environmentally-collected plastic particles (Holmes et al., 2012; Imhof et al., 2016; Massos and Turner, 2017; Wang et al., 2017; Vedolin et al., 2018). Furthermore, degradation of plastics in the environment, e.g., via photooxidation and hydrolysis, generates new sorption sites for uptake of metals (Fotopoulou and Karapanagioti, 2012; Turner and Holmes, 2015). Accordingly, it is anticipated that plastic particles will modify the chemical speciation, bioavailability, and potential toxicity of trace metals in their contact with a given type of water column. Herein we initiate a framework for assessing the potential impact of MP and NP particles on the chemodynamics and bioavailabilities of hydrated trace metal ions, M_aq_, in aquatic media. The focus herein is on the *release* kinetics of metal ions from plastic particles that have been immersed/suspended in an aquatic system for an extended period. Accordingly, the relevant metal species include those that are an inherent component of the original plastic material as well as those that have been sorbed from the aqueous environment. The rate of *association* of metal ions with plastic particles upon their release into aquatic systems will generally follow the framework already elaborated for nanoparticulate complexants (Pinheiro et al., 2005; van Leeuwen and Buffle, 2009; van Leeuwen et al., 2017), albeit that hydrophobic interactions may also play a role.

## Theory

### Physicochemical Features of Plastic Particles in Aqueous Media

Upon their release into aquatic systems, plastic particles will sorb water. Water penetrates polymeric materials by hydrogen-bonding interactions, and different types of polymers hydrate to varying extents. For example, at saturation, PET was found to have a water content of *ca*. 1 water molecule per 10 monomer units, whilst Nylon had *ca*. 6 waters per 10 monomers (Langevin et al., 1994). Furthermore, whilst water molecules sorbed in hydrophobic polymers may be less interacting with each other as compared to that in pure water (Fukuda et al., 1990), hydrogen-bonded water clusters have been proposed as a general feature of hydrophobic plastic matrices (Mountz et al., 2005). Literature data on the diffusion coefficient of water and metal species in various polymer phases are collated in Table 1. For metals, the conditions typically refer to non-acidic aqueous media.

**Table 1:** Literature data on diffusion coefficients of water and metal species in polymer phases.

**Polymer**	**Diffusion coefficient/m**^****2****^ **s**^****−1****^^**a**^	
	**H_**2**_O**	**Metal species (see footnotes for details)**
LDPE	8.4 × 10^−14^ (*T* = 25°C, liquid water)^b^	Pb: 1.5 × 10^−20^ (room *T*, non-structural)^z^
	4.8 × 10^−13^ (*T* = 25°C, water vapor, 85% humidity)^c^	
	6.8 × 10^−12^ (*T* = 25°C, water vapor, 60% humidity)^d^	
	7.42 × 10^−12^ (*T* = 25°C, liquid water)^e^	
	6.7 × 10^−12^ (*T* = 25°C, liquid water)^f^	
	1.2 × 10^−12^ (*T* = 24.5°C, water vapor, 86% humidity)^g^	
	2.5 × 10^−11^ (*T* not stated, water vapor, 96% humidity)^h^	
	2 × 10^−11^ (*T* = 25°C, water vapor)^i^	
PP	5.2 × 10^−12^ (*T* = 25°C, liquid water)^f^	Ni: 5.48 × 10^−15(i)^, 8.57 × 10^−15(ii)^ (*T* = 80°C, structural)^aa^
	2 × 10^−11^ (*T* = 25°C, water vapor)^i^	Zn: 3.58 × 10^−15(i)^, 7.67 × 10^−15(ii)^ (*T* = 80°C, structural)^aa^
	1.9 × 10^−13^ (*T* = 25°C, liquid water)^j^	Sn: 3.94 × 10^−15(i)^, 5.52 × 10^−15(ii)^ (*T* = 80°C, structural)^aa^
		Ca: 5.76 × 10^−16(i)^, 8.95 × 10^−16(ii)^ (*T* = 80°C, structural)^aa^
PVC	1.5 × 10^−12^ (unplasticized, *T* = 25°C, water vapor)^k^	Zn: 3.3 × 10^−12^ (non-structural, plasticized)^ab^
	3.1 × 10^−12^ (plasticized PVC-acetate copolymer, *T* = 25°C, water vapor)^k^	Zn: 4 × 10^−18^, 4 × 10^−17^ (structural, unplasticized, *T* = 30°C)^ac^
	4 × 10^−11^ (plasticized PVC-acetate copolymer, *T* = 20°C, water vapor)^k^	Zn: 1 × 10^−15^ (structural, plasticized, *T* = 30°C)^ac^
		Zn: 4.5 × 10^−18^ (structural, plasticized, *T* = 30°C)^ac^
		Cd: 5 × 10^−20^ (structural, unplasticized, *T* = 30°C)^ac^
		Cd: 8 × 10^−19^ (structural, plasticized, *T* = 30°C)^ac^
		Cd: 1.7 × 10^−17^ (structural, plasticized *T* = 30°C)^ac^
		Pb: 6 × 10^−22(i)^, 1.5 × 10^−21(ii)^, 4 × 10^−23(iii)^, 2 × 10^−22(iv)^ (structural, unplasticized, *T* = 30°C)^ac^
		Pb: 6 × 10^−17(i)^ (structural, plasticized) (*T* = 30°C)^ac^
		Pb: 4 × 10^−18(i)^ (structural, plasticized) (*T* = 30°C)^ac^
		Pb: 5.7 × 10^−18(ii)^ (structural, plasticized) (*T* = 30°C)^ac^
PS	1.2 × 10^−12^ (*T* not specified, liquid water)^l^	
PET	5.6 × 10^−13^ (*T* = 25°C, liquid water)^e^	
	5 × 10^−13^ (*T* = 25°C, liquid water)^f^	
	4 × 10^−13^ (*T* = 25°C, water vapor)^i^	
	6.1 × 10^−13^ (amorphous, *T* = 23°C, water vapor, 0–90% humidity)^m^	
	4.5 × 10^−13^ (amorphous, water vapor and liquid, *T* = 20°C)^n^	
	9.6 × 10^−13^ (amorphous, *T* = 35°C, water vapor)^o^	
	8.18 × 10^−13^ (amorphous, *T* = 25°C, water vapor)^p^	
	1.5 × 10^−14^ (*T* = 35°C)^q^	
	1.4 × 10^−13^ (3.3% crystallinity, *T* = 30°C, water vapor)^r^	
	8.57 × 10^−13^ (4% crystallinity, *T* not stated, liquid water)^s^	
	1.80 × 10^−13^ (16.2% crystallinity, *T* not stated, liquid water)^s^	
	9.97 × 10^−13^ (*T* = 40°C, liquid water)^t^	
Nylon-6	2.2 × 10^−13^ (*T* = 25°C, water vapor, 60% humidity)^d^	Mn: 1 × 10^−14^ (25% crystallinity, non-structural)^u^
	2 × 10^−13^ (*T* = 25°C, liquid water)^f^	
	2.5 × 10^−14^ (*T* = 35°C)^q^	
	1 × 10^−12^ (25% crystallinity, liquid water)^u^	
	1.4 × 10^−12^ (*T* = 25°C, water vapor, water activity 0.9)^v^	
	1.4 × 10^−14^ (*T* = 23°C, water vapor, water activity 0–0.85)^w^	
	4.6 × 10^−13^ (*T* = 25°C, liquid water)^x^	
	6.5 × 10^−13^ (*T* = 25°C, water vapor, water activity 0.9)^y^	

a “Structural” denotes elements that were an inherent component of the polymer matrix; “non-structural” means that the elements were not an inherent component of the polymer matrix; “unplasticized” means no added plasticizing agents; “plasticized” means plasticizing additives are present

b *Wang et al., 2011*;

c *Doty et al., 1944*;

d *Hauser and McLaren, 1948*;

e *Métayer et al., 1999*;

f *Marais et al., 2000*;

g *Penon et al., 2007*;

h *Tong-That and Jungnickel, 1999*;

i *Yasuda and Stannett, 1962*;

j *Yi and Pellegrino, 2002*;

k *Doty, 1946*;

l *Mountz et al., 2005*;

m *Dubelley et al., 2017*;

n *Langevin et al., 1994*;

o *Burgess et al., 2014*;

p *Rueda and Varkalis, 1995*;

q *Schollmeyer et al., 1976*;

r *Fukuda et al., 1990*;

s *Sammon et al., 2000*;

t *Kloppers et al., 1993*;

u *Reuvers et al., 2014: measured Mn diffusion into polymer from aqueous solution of MnCl_2_*;

v *Broudin et al., 2015*;

w *Hernandez and Gavara, 1994*;

x *Monson et al., 2008*;

y *Arhant et al., 2016*;

z *Hnatowicz et al., 1994: measured Pb diffusion into polymer from aqueous solution of Pb(CH_3_COO)_2_*;

aa *Tocháček and Sidlár, 1990: metals are present as complexes with (i) 6-[3-(3,5-di-t-Bu-4-hydroxyphenyl)propanamido]caproic acid or (ii) 3-(3,5-di-t-Bu-4-hydroxyphenyl)propionic acid*;

ab *Chmiel et al., 2003: measured diffusion of Zn into polymer from aqueous solution of ZnCl_2_*;

ac *Mercea et al., 2018: Cd and Zn probably present as stearates, Pb present as (i) mixture of lead stearate and dibasic lead phosphite, (ii) unknown forms, (iii) lead stearate, (iv) dibasic lead phosphite*.

Diffusion coefficients have also been reported for elements that form monovalent ions. For Na^+^, a diffusion coefficient of 1.5 × 10^−13^ m^2^ s^−1^ in Nylon-6 at *T* = 50°C has been reported (Iijima et al., 1978), and the diffusion coefficient for Li^+^, Na^+^, and K^+^ in low water content acrylate and methacrylate polymers is found to be of the order of 10^−13^ m^2^ s^−1^ (Chang et al., 2018).

The range of diffusion coefficients, *D*, reported for H_2_O and metal species reflects differences in crystallinity, plasticizer content and % humidity at which the measurements were made. In the case of water, measurements refer to diffusion of water (vapor) into an initially dry polymer phase; in the case of metal ions, the measurement typically refers to diffusion into or out of the water-saturated polymer (immersed in aqueous solution). The diffusing metal ions are proposed to be at least partially hydrated (Reuvers et al., 2014). The permeation of water into the polymer phase causes a degree of plasticization and a reduction in the glass transition temperature, which in turn can modify the diffusivity of water (Marais et al., 2000; Mountz et al., 2005; Reuvers et al., 2015; Dubelley et al., 2017). Nevertheless, across all polymer types shown in Table 1, the effective diffusion coefficient *D*_eff_ for water lies in the range 10^−11^ to 10^−14^ m^2^ s^−1^. In contrast, a much greater range of *D*_eff_ values is found for the metal species. This range reflects the nature of the diffusing species, i.e., the free metal ion and/or small complexes that are mobile in the polymer phase, and the strength of the interactions with immobile sites on the polymer backbone (see following section).

### Release Kinetics of Metal Ions From Plastic Particles

The data collated in Table 1 show that the effective diffusion coefficients for metal species in polymer phases are orders of magnitude lower than those for diffusion of free hydrated metal ions in bulk aqueous media (typically *ca*. 8 × 10^−10^ m^2^ s^−1^; von Stackelberg et al., 1953). For the case in which metal species are situated within the body of the plastic particle (whether being inherent components of the polymer matrix or acquired from the surroundings), the problem thus reduces to evaluating the release kinetics for the case where diffusion within the particle body is the rate-limiting step. The basic mathematical framework for this case has been developed by Crank (1979). The leading conservation equation for diffusive release of metal, M, from a spherical body of radius *r*_p_ into the surrounding medium is:

(1) $$\begin{array}{r} \frac{\partial c_{\text{M}\left(r,t\right)}}{\partial t}=D_{\text{eff}}\left[\frac{\partial^{2}c_{\text{M}\left(r,t\right)}}{\partial r^{2}}+\frac{2}{r}\left(\frac{\partial c_{\text{M}\left(r,t\right)}}{\partial r}\right)\right] \end{array}$$

where *r* is the distance from the center of the particle, *c*_M(*r, t*)_is the concentration of metal species inside the particle at position *r* and time *t*, and *D*_eff_ is the effective diffusion coefficient for M inside the particle. If the relevant diffusing species are the free metal ions whereas M is immobile during the time of its association with reactive sites in the polymer backbone, then *D*_eff_ is simply given by the pertaining diffusion coefficient of the free M:

(2a) $$\begin{array}{r} D_{\text{eff}}=\frac{D_{\text{M},\text{f}}c_{\text{M},\text{f}}}{c_{\text{M},\text{t}}}\;[{\text{m}}^{\text{2}}{\text{s}}^{-\text{1}}] \end{array}$$

where D_M,f_ is the diffusion coefficient for the free metal ion within the particle body (as defined by diffusion in the hydrated zones and/or inside the polymer phase), and *c*_M,f_ and *c*_M,t_ are the respective concentrations of the free metal ion and the total M within the particle. If the diffusing species also includes mobile and labile metal complexes, ML, where L is e.g., a stabilizer present in the polymer phase (as applicable for some of the data in Table 1) then *D*_eff_ represents the mean diffusion coefficient of the free M and the complex ML:

(2b) $$\begin{array}{l} D_{\text{eff}}=\frac{D_{\text{M},\text{f}}c_{\text{M},\text{f}}+D_{\text{ML}}c_{\text{ML}}}{c_{\text{M},\text{t}}}\;[{\text{m}}^{\text{2}}{\text{s}}^{-\text{1}}] \end{array}$$

where *D*_ML_ and *c*_ML_ are the respective diffusion coefficient and concentration of mobile metal complexes in the polymer phase. The ratio *c*_M,f_/*c*_M,t_ (Equation 2a) or (*c*_M,f_ + *c*_ML_)/*c*_M,t_ (Equation 2b) represents the mobile fraction of intraparticulate M. Equation (2) assumes that the free and complexed forms of M are in equilibrium with each other, i.e., at the particle/medium interface ML is labile on the timescale of the diffusive process. This condition amounts to the rate constant for dissociation of the complex species, $k_{\text{d}}^{\text{is}}$, being sufficiently high to outweigh the corresponding rate constant for diffusion inside the polymer phase. The magnitude of $k_{\text{d}}^{\text{is}}$ is determined by the thermodynamic stability of the metal complex and the inner-sphere dehydration rate constant of the metal ion.

Equation (1) is solved under the initial and boundary conditions pertaining to (i) randomly distributed M in the particle body in the starting situation of the release process, and (ii) insignificance of the fast diffusion inside the external medium, such that the concentration of M at the medium side of the particle/medium interface is essentially zero (limiting flux). The latter condition requires a sufficiently low volume fraction of plastic particles. These conditions correspond to:

initial condition:

(3a) $$\begin{array}{r} t=0:\;0<r<r_{\text{p}}:c_{\text{M}}=c_{\text{M}}^{\text{p}};\;r>r_{\text{p}}:\;c_{\text{M}}^{\text{aq}}=0 \end{array}$$

where $c_{\text{M}}^{\text{p}}$ is the initial uniform (smeared-out) concentration of M inside the particle and $c_{\text{M}}^{\text{aq}}$ is the concentration of M in the surrounding bulk aqueous medium.

boundary condition:

(3b) $$\begin{array}{r} t>0;\;r=r_{\text{p}}:c_{\text{M}}^{0}=0 \end{array}$$

with $c_{\text{M}}^{0}\left(r=r_{\text{p}}\right)$ being the concentration of M at the particle surface.

The solution for Equation (1) with boundary conditions (3a) and (3b) is (Crank, 1979):

(4) $$\begin{array}{r} \frac{\text{M}\left(t\right)}{{\text{M}}_{\left(t\to \infty \right)}}=1-\left(\frac{6}{\pi^{2}}\overset{\infty }{\underset{n=1}{\sum }}\frac{1}{n^{2}}\exp\left[-n^{2}\pi^{2}\left(t/\tau \right)\right]\right) \end{array}$$

where M(*t*) is the *amount* that has been released from the spherical particle at time *t*, M_(*t*→∞)_is the amount that has been released at infinite time, and τ is the fundamental time constant (= $r_{\text{p}}^{2}/D_{\text{eff}}$) for diffusive release from the particle. Equation (4) is used to predict the extent to which inherent and sorbed M is released from the body of MPs and NPs.

For the case of metal ions that are present at the particle surface, i.e., sorbed at the particle/water interface or sorbed by surface-sorbed ligands such as natural organic matter, the release process does not involve diffusion through the polymer phase. For metal ions that are sorbed to the surface of the polymer matrix, the diffusion-controlled rate constant, *k*_d,p_, for dissociation is given by (van Leeuwen et al., 2017):

(5) $$\begin{array}{r} k_{\text{d},\text{p}}=3D_{\text{M}}{\bar{f}}_{\text{el},\text{d}}\left(1+K_{\mathrm{int}}^{\text{2D}}\Gamma_{\text{S}}\right)/r_{\text{p}}^{2}\;\left[s^{-1}\right] \end{array}$$

where *D*_M_ is the diffusion coefficient for the metal ion in bulk aqueous medium, ${\bar{f}}_{\text{el},\text{d}}$ is the electrostatic coefficient for conductive diffusion away from the particle (see van Leeuwen et al., 2017 for details), $K_{\mathrm{int}}^{\text{2D}}$ (m^2^ mol^−1^) is the intrinsic stability constant for inner-sphere complexes between the metal ion and the surface sorption sites, and Γ_S_ is the surface concentration of binding sites (mol m^−2^). The intrinsic stability constant represents the inherent chemical affinity between a metal ion and a reactive site, after correction for the long-range electrostatics beyond those on the scale of atom–atom interactions (Town and van Leeuwen, 2016). For metal ions that are associated with a permeable surface coating of e.g., natural organic matter or other complexants, *k*_d,p_is given by (van Leeuwen et al., 2017):

(6) $$\begin{array}{r} k_{\text{d},\text{p}}=3D_{\text{M}}{\bar{f}}_{\text{el},\text{d}}\left(1+K_{\text{int}}^{3\text{D}}c_{\text{S}}\right)/r_{\text{p}}^{2}\;\left[s^{-1}\right] \end{array}$$

where $K_{\text{int}}^{3\text{D}}$ (m3 mol^−1^) is the intrinsic stability constant for inner-sphere complexes between the metal ion and the binding sites within the permeable coating and *c*_S_ is the concentration of binding sites within the permeable coating (mol m^−3^).

In the general case of plastic particles in environmental media, the applicable *K*_int_ values and the concentrations of binding sites are unknown. However, for the limiting case in which the intrinsic binding affinity becomes immaterial, e.g., in an acidic gut environment, the applicable *k*_d,p_ is the conventional one for diffusion from an uncharged, non-complexing sphere (Zhang et al., 2007; van Leeuwen et al., 2012):

(7) $$\begin{array}{r} k_{\text{d},\text{p}}=3D_{\text{M}}/r_{\text{p}}^{2}\;\left[s^{-1}\right] \end{array}$$

It is pertinent to note that the model of a randomly filled sphere might be a poor model for an arbitrary plastic particle in the environment. Development of a more sophisticated interpretation framework is hindered by the current lack of quantitative information on the pore structure within the particle body, the thermodynamic and kinetic features of metal-polymer species, *etc*. Until such details are at hand, our simple sphere model provides an order-of-magnitude estimate of the characteristic time of release of M from plastic particles, and this may serve as an important starting point for future work.

## Results and Discussion

### Predicted Diffusion Limited Release Kinetics of M From Plastic Particles

**Figure 1:**
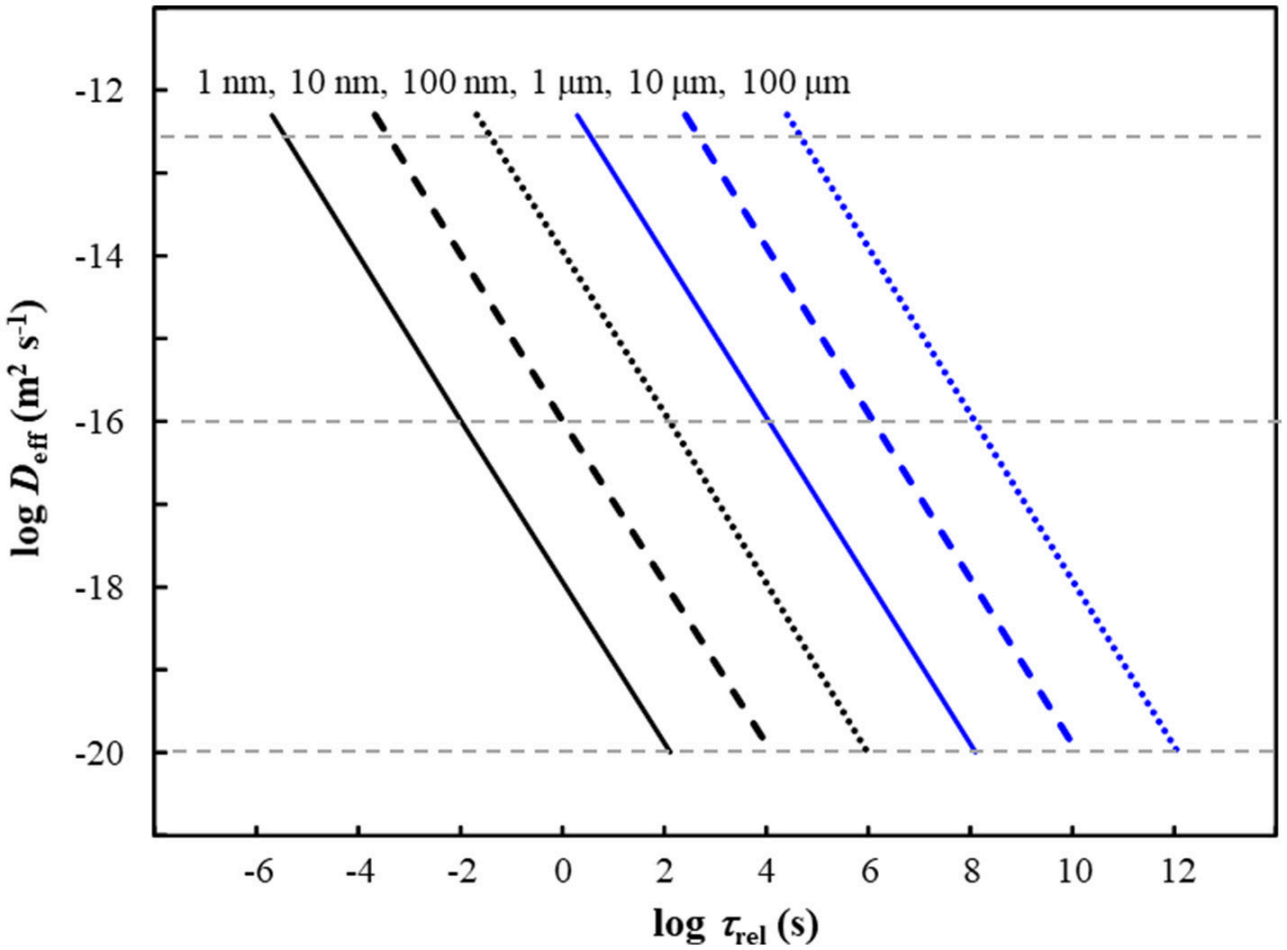
Typical time, τ_rel_, required for complete release of M from plastic particles as a function of the effective diffusion coefficient *D*_eff_ (*y*-axis) and the particle radius, *r*_p_ (indicated above each line). Typical values of *D*_eff_ reported for M in polymers are indicated by horizontal gray dashed lines (see Table 1).

**Figure 2:**
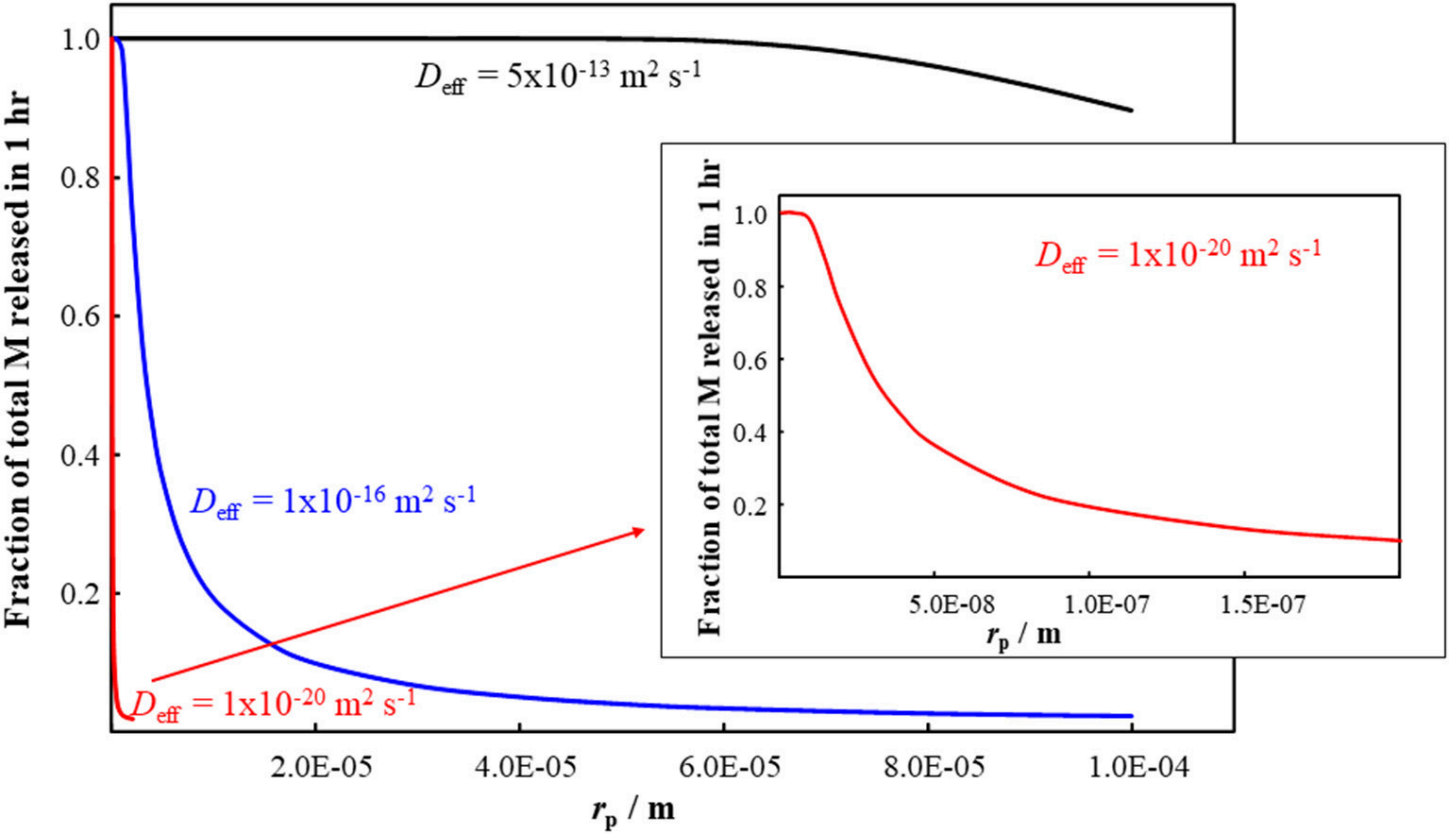
Fraction of total M released from a plastic particle in 1 h as a function of the particle radius, *r*_p_, and the effective diffusion coefficient, *D*_eff_, values indicated on the figure. The inset is an amplification of the result for *D*_eff_ = 10^−20^ m^2^ s^−1^.

Since the diffusion coefficient for M species within the plastic matrix is much smaller than that in bulk water (Table 1), we apply Equation (4) to predict the release kinetics of M species from within the body of plastic particles. The applicable *D*_eff_ is determined by the fraction of the total M that is present in the form of free ions and mobile complexes within the polymer phase (Equation 2). The typical timescale, τ_rel_, required for the complete release of M from nano- and micro-plastic particles is shown in Figure 1 as a function of particle size (*r*_p_) and intraparticulate mobility (*D*_eff_) of the species of M. The figure indicates that, over the timescale of 1 h, M species with a *D*_eff_ greater than order of 10^−20^ m^2^ s^−1^ would be completely released by small plastic nanoparticles (*r*_p_ up to order 10 nm). The fraction of the total M in the plastic particles that would be released within 1 h is shown in Figure 2 as a function of the particle radius and *D*_eff_. The results show that metal species with a *D*_eff_ of 5 × 10^−13^ m^2^ s^−1^ would be practically completely released within 1 h from particles with *r*_p_ up to order 100 μm. In contrast, over the 1 h timescale, significant release of M species for which *D*_eff_ is of order 10^−16^ m^2^ s^−1^ or less is only expected for particles in the nanosize regime. These results fit with *ad hoc* observations of increased release of M from plastics as the size of the particles is decreased (Fowles, 1977; Wilson et al., 1982).

### Application of Theory to Describe Release Kinetics of M From Plastic Particles

The setting of interest is the release of M from plastic particles that have been ingested by biota. The pH varies along the digestive tract according to the involved compartment and the organism's physiology. The stomach, or equivalent digestive compartment, is generally acidic. Furthermore, intracellular digestion within acidic vesicles such as lysosomes can be an important process in some organisms. Notably, microplastics have been shown to accumulate in the lysosomal system of mussels (van Moos et al., 2012). In acidic media, any complexes of M, e.g., those added as stabilizers as well as those between M and immobile sites on the polymer backbone, will tend to dissociate. That is, the concentration of free M in the polymer phase, *c*_M,f_, and the ensuing *D*_eff_ (Equation 2), is expected to be greater than that operative for equivalent MPs in the bulk aqueous medium at circumneutral pH. Accordingly, ingestion of plastic particles may lead to localized release of metal ions into certain tissues and intracellularly via lysosomes.

For plastic particles in aquatic ecosystems, the amount of associated metal ions will vary with the type of polymer and associated additives as well as the conditions prevailing at a given location. For example, using a short term (2 h) extraction of MPs with 10% nitric acid, the highest level of extractable Cd amongst a diverse range of beach collected MPs was found to be 0.93 mg g^−1^ from a PVC particle (Munier and Bendell, 2018). Estimation of metal availability from ingested particles typically involves use of physiologically-based extractions, e.g., acidic enzymatic solutions (Oomen et al., 2002). In the case of MPs, typically only a small fraction of the total M content is released over the timescales considered. For example, a 7 day physiologically-based extraction of beach collected MPs was found to release *ca*. 2% of the total Cd content (Massos and Turner, 2017). The authors did not report the size distribution of the MPs, but only particles that were visible to the naked eye were collected, and the reported average mass of *ca*. 35 mg per particle suggests that the particles were of mm dimensions. Thus, the low extractability of Cd is in line with our computations: Figure 1 shows that for *r*_p_ of 0.1 mm, only the M with *D*_eff_ of order 10^−12^ m^2^ s^−1^ would be released after 7 days (*ca*. 10^6^ s).

**Figure 3:**
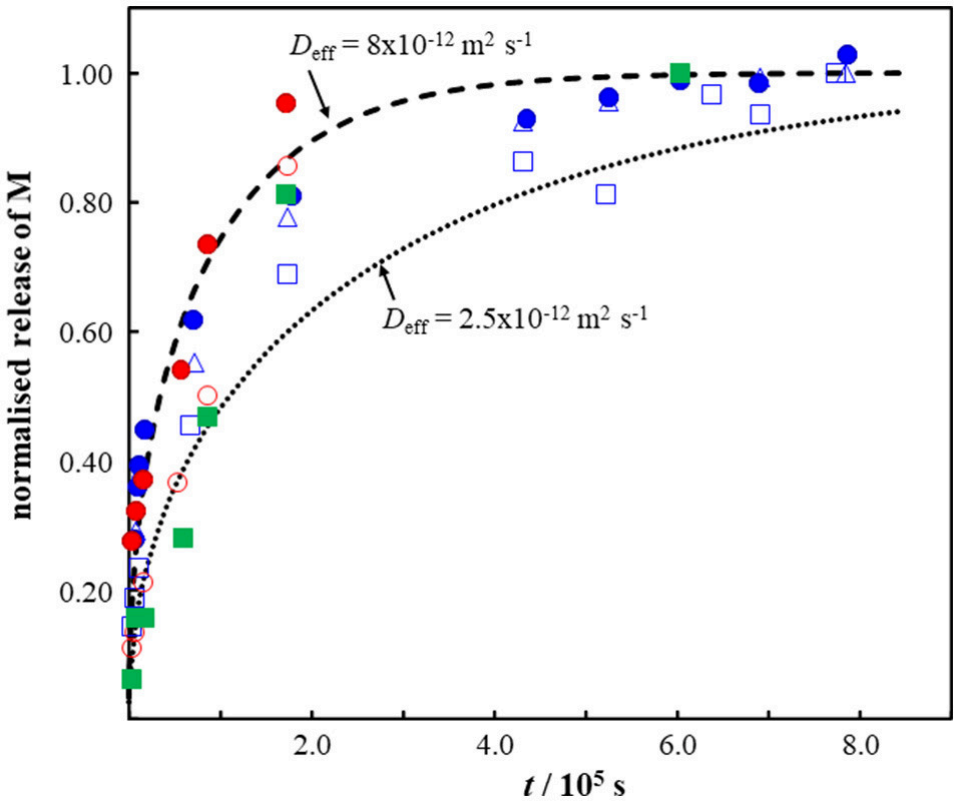
Comparison of experimentally measured (points) (Turner and Lau, 2016; Turner, 2018) and computed (dashed curves) release of Pb (blue symbols) and Cd (green and red symbols) as a function of time from fragments of beach-collected plastics. The dashed curves were computed using Equation (4) with particle radius = 3 × 10^−3^ m and the *D*_eff_ values indicated on the figure. The plastic samples correspond to *ca*. 0.1 g “shavings” of polyurethane (blue symbols), polypropylene (red symbols), and polyethylene (green symbol). See text for details.

The applicability of our simple sphere model to describe the time course of M release from MPs is illustrated by use of data for Pb and Cd release from irregular fragments (“shavings” of *ca*. 0.1 g) of beach-collected polyurethane (Turner and Lau, 2016), polypropylene and polyethylene (Turner, 2018). The MPs were extracted with a model gut solution (pepsin, pH 2.5, 40°C) for 220 h (Turner and Lau, 2016; Turner, 2018). Figure 3 compares the reported time course of M release with that predicted by Equation (4). The “shavings” of 0.1 g were modeled as a sphere with *r*_p_ of 3 × 10^−3^ m, and an effective diffusion coefficient in the range 2.5 × 10^−12^ to 8 × 10^−12^ m^2^ s^−1^ provides a reasonable description of the data (Figure 3). The magnitude of *D*_eff_ suggests that the diffusing species is the (partially) hydrated metal ion (*cf*. data in Table 1). Furthermore, this finding indicates that the amount of M that is surface sorbed, or associated with surface sorbed complexants, must be negligible with respect to that which is located within the particle body. The applicable diffusion coefficient for surface-associated M is that for the free M in the bulk aqueous medium, Equation (7), i.e., *ca*. two orders of magnitude greater than the observed value. The authors reported that up to *ca*. 10% of the total M content of the MPs was extractable within 220 h (Turner and Lau, 2016; Turner, 2018). This outcome implies that the proportion of M that is not extracted remains associated with immobile sites on the polymer backbone and/or in the form of complex species that have a diffusion coefficient orders of magnitude lower than that for the free M in the polymer phase (Table 1). In the latter case, the timescale of diffusive release of free M may be separated from that of the mobile complexes even if the system is fully labile (van Leeuwen, 2011).

### Potential Bioavailability of Metals Associated With Micro- and Nano-Plastics

There are conflicting reports in the literature concerning the effect of plastic particles on the bioaccumulation and potential toxicity of metals. Furthermore, many studies do not report the extent of metal-particle association in the exposure medium, and thus meaningful interpretation is impossible (Davarpanah and Guilhermino, 2015; Barboza et al., 2018). The results discussed in the preceding sections highlight that the potential bioavailability of metals associated with plastic particles will depend on their physicochemical forms in the particle body, the size of the particles, as well as the timescale of the exposure.

As an illustrative example, we consider a laboratory based study on the effect of spherical polyethylene MPs (*r*_p_ = 30 μm) on the uptake of Ag by adult zebrafish (wet weight *ca*. 0.5 g) (Khan et al., 2015). The results showed that in the presence of Ag-MP entities, the Ag content of intestinal tissues after 4 h exposure was approximately a factor of 10 greater than that found following exposure to Ag^+^ only (at the same total Ag concentration of 9.27 × 10^−6^ mol m^−3^) (Khan et al., 2015). In the presence of the MPs, 76% of the total Ag was associated with the particles. The Ag in this case was not an initially inherent component of the MP and the applicable *D*_eff_ will depend on the strength of the sorptive binding of Ag to the polymer backbone (Equation 2). For the given size of MP (*r*_p_ = 30 μm), our computations predict that Ag with a *D*_eff_ of order 10^−12^ m^2^ s^−1^ would be completely released within 1 h, whilst if the *D*_eff_ is of order 10^−16^ m^2^ s^−1^ then <10% would be released in this timeframe (Figures 1, 2). For the given exposure conditions, the reported Ag content in the intestinal tissues would correspond to the total Ag content of 868 MPs per fish. Although the authors did not attempt to determine the presence of MPs in the intestinal tissue (Khan et al., 2015), their ingestion is expected given that the fish were starved for 24 h prior to exposure and the MP content in the medium was 10^3^ particles per mL. It is noteworthy that the total Ag body burden of the zebrafish in this short term exposure was reduced in the presence of MPs (by a factor of *ca*. 2.5) (Khan et al., 2015). In another study, a longer term, 3 week exposure of Cd and MPs to zebrafish revealed changes in the tissue distribution of accumulated Cd in the presence of MPs, as well as differences in biochemical markers and gene expression (Lu et al., 2018). Overall these observations suggest that the uptake route and timescale of accumulation of metal species associated with MPs, as well as the subcellular compartmentalization of such metal species, can differ substantially from that for free aqueous metal ions. It can be anticipated that nanoparticulate plastics will have even more dramatic effects on the chemical and biological reactivity of associated metal species (van Leeuwen et al., 2013, 2017); Figures 1, 2.

## Conclusions and Outlook

Plastics have a very long residence time in the environment and contain a metal load in the form of additives and environmentally acquired metals. The association of metal ions with plastic particles in aquatic systems will modify their speciation dynamics, and in turn their potential bioavailability. The association of metal species with plastic particles alters the spatial scale and timescale of the external and internal exposure conditions in a manner that depends on the physicochemical features of the particles, notably *r*_p_, as well as the physiology of the organisms, e.g., feeding behavior, gastric digestion, and gut retention times. Nanoparticle sized plastics may be of particular concern because all forms of M associated with the particle body are expected to be released within a short timescale in acidic gut and lysosomal environments. Such local bolus release of metal ions may trigger toxic effects. Nevertheless, even if metal species associated with plastic particles at typical environmental concentrations do not cause acute toxicity, they may well alter the timescale of exposure and the sub-cellular compartmentalization of metal species within organisms, resulting in subtle and long-term detrimental effects on ecosystems.

For environmental risk assessment purposes, quantitative data is needed on the amount and physicochemical speciation of metal ions associated with micro- and nano-plastic particles. This information, together with knowledge on MP and NP ingestion rates and other physiological factors such as gut transition times, can be used to estimate the potential significance of plastic particles as vectors for metal exposure, as compared to metal exposure via the aqueous phase and diet. Particularly for filter feeders, e.g., mussels, metals released from ingested plastic particles may be significant as compared to metal exposure via the water phase. For example, consider a typical marine exposure scenario for Cd(II) in which the concentration of aqueous Cd(II) is *ca*. 0.1 nM (WHO, 1992; OECD, 1994) and *micro*plastics are present in the range 1–100,000 particles per m^3^ (Enders et al., 2015; Auta et al., 2017). Approximately 20% of the MPs collected in one study (Enders et al., 2015) had an *r*_p_ of order 5 μm which is similar to the size of dietary particles ingested by mussels. A mussel with a filtration rate of order 2 L per hour per individual (Yakan et al., 2011) would be exposed to 0.2 nM of Cd(II) per hour via the aqueous phase and potentially 10^−5^ to 1 nM per hour via ingested MPs (assuming that 20% of the MPs have an *r*_p_ of order 5 μm and a Cd content of 1% w/w which is completely released). This simple estimate ignores the potential metal exposure that may derive from ingestion of *nano*plastic particles. As detailed herein, the amount of M released in practice from ingested particles will depend on the size of the particles, the assimilation efficiency, the prevailing gut conditions and the transit time through the digestive tract. The significance of metal exposure via plastic particles as compared to other ingested organic and inorganic particles will be highly variable.

Whilst the present context is release of M from ingested particles, the concepts are generically applicable to the chemodynamic features of metal species associated with plastic particles. The outcomes of our work are also significant for analyses of the allowed migratabilities of metals in plastic consumer goods, e.g. toys. Current test procedures for migration of compounds in polymeric materials specify sample dimensions of *ca*. 6 mm (European Committee for Standardization, 2018); evidently the actual migratable amount of metals, ánd the timescale of their release, will be strongly dependent on the size of the particles (Fowles, 1977; Wilson et al., 1982).

The simple model presented herein may serve as an educated starting point for predicting the chemical reactivity and potential bioavailability of metals associated with spherical plastic particles, and can be adapted to different particle geometries. Development of a more quantitative interpretation framework is hampered by the lack of information on the physicochemical features of polymeric particles, e.g., internal pore structure, and the thermodynamic stability and kinetic features of M-polymer associates. Future work will develop a comprehensive model for the chemodynamics and bioavailability of metal species associated with plastic particles that links M release kinetics to the ensuing bioaccumulation and subcellular distribution of metal species.

## Author Contributions

RT collated the source literature data. RT and HvL drafted the manuscript and formulated the modeling framework. RB critically reviewed the manuscript.

### Conflict of Interest Statement

The authors declare that the research was conducted in the absence of any commercial or financial relationships that could be construed as a potential conflict of interest.
